# A weekday intervention to reduce missed appointments

**DOI:** 10.1371/journal.pone.0274670

**Published:** 2022-09-29

**Authors:** David A. Ellis, Jet G. Sanders, Rob Jenkins, Linda McAuslan

**Affiliations:** 1 School of Management, University of Bath, Bath, United Kingdom; 2 Department of Psychological and Behavioural Science, London School of Economics and Political Science, London, United Kingdom; 3 Department of Psychology, University of York, York, United Kingdom; 4 NHS Forth Valley, Psychology Department, Mayfield Building, Falkirk, United Kingdom; University of South Australia, AUSTRALIA

## Abstract

The burden of missed healthcare appointments is so great that even small reductions in Did Not Attend (DNA) rate can secure tangible benefits. Previous studies have identified demographic factors that predict DNA rate. However, it is not obvious that these insights can be used to improve attendance, as healthcare providers do not control patient demographics. One factor that providers do control is appointment scheduling. We previously reported that appointments at the beginning of the week are more likely to be missed than appointments at the end of the week. This observation suggests a simple intervention to reduce DNA rate: schedule appointments for later in the week. Using data from a UK mental health hospital, we compared attendance rates for 12-months before and 12-months after the intervention began (916 appointments in total). Overall DNA rate fell from 34.2% pre-intervention to 23.4% post-intervention [χ2 (1, *N* = 916) = 13.01, *p* < 0.001; Relative Risk Reduction, 31.6%]. This effect was carried mainly by female patients, for whom more appointments could be moved to later in the week. Our findings confirm that DNA rate can be significantly reduced by loading appointments onto high-attendance days.

## Background

Missed medical appointments are a long-standing problem for patients and for health service providers [1]. Clinical intelligence shows that missing appointments is a strong predictor of poor health outcomes, particularly among in-patient groups with high levels of deprivation, and those suffering from multiple long-term conditions [2]. Non-attendance also represents a major inefficiency in the healthcare system [3]. For these reasons, there is widespread interest in improving attendance rates. Progress on this front requires a clear understanding of why people miss medical appointments. Although many contributing factors have been put forward, these factors are often highly context-specific, relying on small samples from different clinical settings [4, 5]. While these results may be informative, they do not point towards a widely applicable intervention that might increase attendance across multiple clinics in primary and secondary care. To date, successful interventions have focused on text-message or letter reminders [6, 7], which can be costly to implement [7].

In recent years, researchers have turned to large data sets to gain new insights into missed appointments and their causes. Analyses of large data have assisted in both confirming previous clinical intelligence and replicating exploratory findings on a larger scale. Some have gone so far as to suggest interventions that could encourage future attendance [8, 9]. For example, while some specific patient populations appear to be at a greater risk of missing appointments, (e.g. patients with multiple mental health diagnoses [2]), several patterns of non-attendance appear stable across multiple patient groups. For example, missed appointment rates are generally higher for patients who are male, or young, or low socioeconomic status [8]. Such patterns can therefore be used to inform future intervention strategies or develop information systems that support patients as they move through a healthcare system [8].

However, here we take a different approach that focuses on appointment scheduling rather than on patient demographics. Recent studies have shown that the day of the week on which an appointment is held can affect attendance rate for those appointments [9–11]. The largest of these studies [9] analysed over 4 million outpatient appointments. Did Not Attend (DNA) rate was highest for Mondays, lowest for Fridays, and decreased monotonically over the week. This decline in DNA was present for male and female patient groups in all age groups, and was steeper for younger patients.

Similar weekday effects have now been shown in different types of medical appointments, from General Practice (GP) visits and scheduled hospital visits [9] to specialist surgical operations [10, 11]. Weekday effects are typically small compared with demographic effects [9]. Importantly however, appointment allocation policy can be changed easily, whereas the gender, age, and socioeconomic status of patients can not. For these reasons, weekday effects may be of greater practical significance despite their smaller size. Simply scheduling appointments for later in the week could reduce non-attendance across demographic categories. This approach should also be inexpensive compared with other interventions, as it does not require any additional equipment or procedures (cf. telephone or text reminders [7]).

## Current study

Following knowledge-exchange activities that included a workshop presentation delivered by the Quality and Efficiency Support Team (QuEST), we had the opportunity to test our recommendation in a clinical setting (a community mental health clinic) in Scotland.

As the expected rate of missed appointments is highest on Mondays and lowest on Fridays [9], the optimal strategy would be to replace all Monday appointments with Friday appointments. That was not possible in this study due to practical constraints. However, the observed decline in missed appointments through the week is monotonic [9]. This implies that any overall change in the distribution of appointments from earlier in the week to later in the week should improve overall attendance.

A Senior Charge Nurse (author LM) responsible for outpatient appointments strategically allocated appointments as late in the week as possible. The total clinical time available each week remained consistent, so changes were straightforward. At the same time, staff had become frustrated at the number of missed appointments and understood the rationale for changes, having been briefed on previous research. Which patients received appointments on earlier versus later weekdays was randomised.

In this particular intervention, the biggest changes were a reduction in Monday allocations (-24.1% year on year) and an increase in Wednesday and Friday allocations (+91.2% and +21.2% year on year, respectively). Given that attendance on Wednesdays and Fridays has previously been found to be higher than attendance on Mondays [7, 9, 10], we expected overall attendance to improve following this intervention. Table 1 shows the distribution of scheduled appointments across weekdays for 12 months before and 12 months after this intervention.

**Table 1:** Summary of the weekday intervention. Separate sections show *Female* patients (top), *Male* patients (middle), and *All* patients (bottom). For each section, the first row (*Before*) shows the distribution of appointments across weekdays Monday to Friday for the 12 months before the intervention. The second row (*After*) shows the same distribution for the 12 months after the intervention. The third row (*Change [%]*) shows the *Before–After* differences as number of appointments and percentages. After the intervention, appointments were loaded onto higher-attendance days. This change was more pronounced for female patients than for male patients.

***Female***	**M**	**T**	**W**	**Th**	**F**	**Total**
*Before*	104	27	35	62	29	257
*After*	68	28	54	63	38	251
*Change [%]*	-36 [-34.6]	+1 [+3.7]	+19 [+54.3]	+1 [+1.6]	+9 [+31.0]	-6 [-2.3]
***Male***	**M**	**T**	**W**	**Th**	**F**	**Total**
*Before*	66	21	22	56	23	188
*After*	61	26	55	53	25	220
*Change [%]*	-5 [-7.6]	+5 [+23.8]	+33 [+150.0]	-3 [-5.4]	+2 [+8.7]	+32 [+17.0]
***All***	**M**	**T**	**W**	**Th**	**F**	**Total**
*Before*	170	48	57	118	52	445
*After*	129	54	109	116	63	471
*Change [%]*	-41 [-24.1]	+6 [+12.5]	+52 [+91.2]	-2 [-1.7]	+11 [+21.2]	+26 [+5.8]

### Analysis

The Integrated Community Mental Health Services (via author LM) provided anonymised records of outpatient appointments from 1^st^ September 2014 to 31^st^ August 31^st^ 2016 (916 appointments in total). IRB approval was not required for the study due to it being regarded as a service evaluation.

The data specified the total number of appointments attended and missed, categorised by weekday, for each month of the 2-year period. A missed appointment was defined as failure to attend having not made contact ahead of time. Appointments were further split into male and female patients. To preserve anonymity, age data were not included. Our main interest was in comparing attendance rates before and after the intervention.

## Results

**Fig 1:**
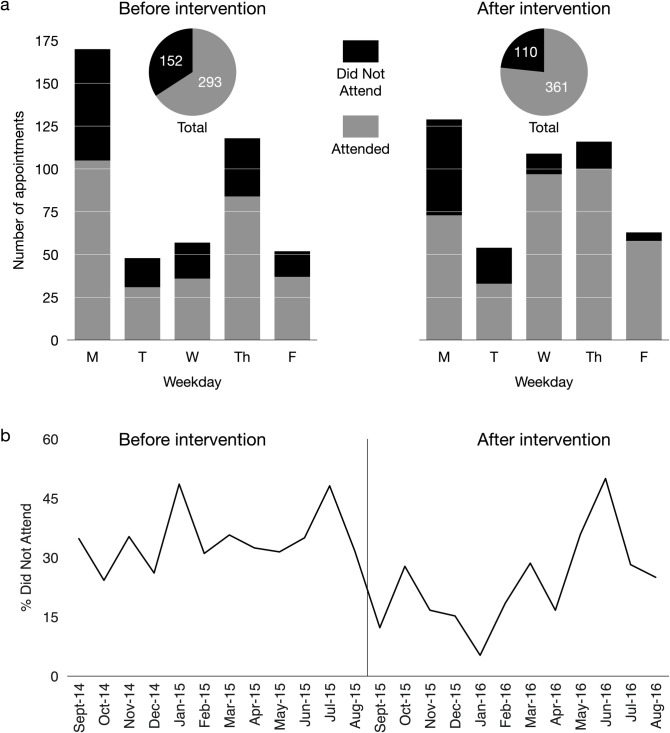
Attendance rates before (left) and after (right) the weekday intervention. a. Redistributing appointments across weekdays improved attendance. b. Time series showing DNA rates before (left) and after the intervention (vertical line). Overall DNA rates were lower post-intervention than pre-intervention.

As with previous studies, appointments at the beginning of the week were more likely to be missed than appointments at the end of the week (see Fig 1A and Table 2). This pattern illustrates the rationale for the weekday intervention.

**Table 2:** DNA rates for each weekday before and after the intervention. Separate sections show *Female* patients (top), *Male* patients (middle), and *All* patients (bottom). For each section, the first row (*Before [%]*) shows the number and percentage of appointments missed Monday to Friday for the 12 months before the intervention. The second row (*After [%]*) shows the corresponding distribution for the 12 months after the intervention.

***Female***	**M**	**T**	**W**	**Th**	**F**
*Before [%]*	45 [43.3]	11 [40.7]	13 [37.1]	17 [27.4]	10 [34.5]
*After [%]*	28 [41.2]	11 [39.3]	4 [7.4]	5 [7.9]	3 [7.9]
***Male***	**M**	**T**	**W**	**Th**	**F**
*Before [%]*	20 [30.3]	6 [28.6]	8 [36.4]	17 [30.4]	5 [21.7]
*After [%]*	28 [45.9]	10 [38.5]	8 [14.5]	11 [20.8]	2 [8.0]
***All***	**M**	**T**	**W**	**Th**	**F**
*Before [%]*	65 [38.2]	17 [35.4]	21 [36.8]	34 [28.8]	15 [28.8]
*After [%]*	56 [43.4]	21 [38.9]	12 [11.0]	16 [13.8]	5 [7.9]

More importantly, the overall DNA rate fell from 34.2% in the 12 months before the intervention to 23.4% in the 12 months after [χ^2^ (1, *N* = 916) = 13.01, *p* < 0.001; Relative Risk Reduction, 31.6%]. Among female patients, this reduction was large (37.6% vs 20.3%) and statistically significant [χ^2^ (1, *N* = 508) = 17.92, *p* < 0.001]. Among male patients, the reduction was smaller and was not statistically significant (29.8 vs 26.8%) [χ^2^ (1, *N* = 408) = 0.44, *p* = 0.51].

Both the pre-intervention year and the post-intervention year show substantial variability over the months. A Chow test for a structural break in the time series revealed a significant difference pre- versus post-intervention [*F*(2, 20) = 6.69, *p* = 0.006] (Fig 1B). This finding localises the change in attendance rates to the time of the intervention. As with the frequency analysis, this effect was carried mainly by female patients [*F*(2, 20) = 5.43, *p* = 0.01] rather than male patients [*F*(2, 20) = 2.72, *p* = 0.09].

## Discussion

Missed appointments were much less common in the year after the intervention, compared with the year before. This improvement was predicted from our analyses of hospital and GP attendance data [9]. The new study goes beyond previous work by demonstrating a successful prospective intervention—preferentially loading appointments towards the end of the week. In the current dataset, the introduction of this policy was immediately followed by a 30% reduction in missed appointments.

Several features of these findings strike us as interesting. First, the rationale for the weekday intervention was based on analysis of data from specific healthcare settings (hospital and GP), while the predicted benefit was detected in a very different healthcare setting (a community mental health clinic). The observation that weekday effects cross settings in this way suggests that they may be broadly applicable across health services. Specifically, the intervention reported here was brought to the attention of nurses following a workshop that considered complex and simple approaches to reduce DNAs. We would encourage further knowledge-exchange activities in this regard, which can occur at any stage of the research process [12].

Second, the observed effect was large (>30% reduction in missed appointments), even though this particular weekday intervention was not especially strong. In the analysis that motivated this intervention [9], the rate of missed appointments was highest on Mondays, lowest on Fridays, and declined monotonically through the week. Given this pattern, any net shift in appointments from earlier in the week to later in the week should deliver some benefit. However, the optimal intervention would be to move Monday appointments to Fridays. In this intervention, the overall shift was mainly from Mondays to Wednesdays. Nevertheless, the observed benefit in DNA rates was substantial. Stronger interventions may deliver larger benefits still. The analysis by gender is potentially informative in this respect. We note that the redistribution of appointments was closer to optimal for female patients than for male patients (see Table 1); and the observed improvement in attendance rate was much greater for female patients than for male patients. One interpretation of this pattern is that stronger interventions deliver larger benefits. Another possibility is that females are more responsive than males to weekday interventions. A full understanding of this pattern likely involves factors that go beyond the available data. For example, logistic factors are also known to affect attendance rates, as when unforeseen work or caring obligations interfere with scheduled appointments [13].

Third, the long-term stability of the intervention effect is unknown. Although we saw an overall drop in DNA rates in the year after the intervention, this drop was not present in every month. In three of the twelve calendar months (October, May, and June), post-intervention DNA rates exceeded pre-intervention rates. Future studies could more cleanly separate intervention effects from background variability by collecting data for several years before and several years after the intervention. Converging evidence from different studies at different sites would establish the generality of the effects.

Estimates for the cost of missed appointments vary [3]. But there is broad agreement that the human cost [2] and the financial cost [3] are both high. The observed 30% reduction in missed appointments implies a 30% reduction in the associated costs. While other methods have achieved similar reductions in the rate of missed appointments, some interventions are themselves costly to implement. For example, automated text message or telephone reminders incur setup and operating costs. Human-operated reminders incur staff costs. Such implementation costs must be counted against any savings, but are sometimes overlooked. One advantage of the weekday intervention is that it may not incur direct implementation costs, as it only requires the scheduling system that is already in use. Another possible advantage is that weekday interventions and reminder interventions might target independent causes of missed appointments, such as forgetting and motivation. If that is the case, then using both interventions in combination could unlock additive gains.

The mechanisms underlying weekly fluctuations in attendance rates are not yet fully understood. One potentially interesting observation is that the profile of attendance over the week closely follows the profile of emotional responses to weekday cues [9–11, 14]. Specifically, attendance improves as mood improves. On this view, positive mood may provide an emotional buffer that makes medical appointments easier to face. This proposal aligns with the many psychological reasons that patients give for non-attendance (e.g. fear of bad news [15], fear of unpleasant treatment [16], negative relationships with staff [17]). More recent work has begun to track systematic changes in decision making and behaviour over the weekly cycle [18]. We anticipate that this work could shed light on cognitive factors that affect attendance rates. For now, we find that preferentially loading appointments towards the end of the week can improve attendance. We encourage other services to try this simple and inexpensive intervention, and to report their findings.

## Supporting information

S1 Data (XLSX)

## List of abbreviations

DNA: Did Not Attend
GP: General Practice
QuEST: Quality and Efficiency Support Team

## References

[1] Williamson A., Ellis D. A., Wilson P., McQueenie R. and McConnachie A. (2017). Understanding repeated non-attendance in health services—pilot analysis of administrative data and full study protocol. *BMJ Open*, 7(2), e014120 10.1136/bmjopen-2016-014120 PMC5319001 28196951

[2] McQueenie R., Ellis D. A., McConnachie A., Wilson P. and Williamson A. E. (2019). Morbidity, mortality and missed appointments in healthcare: a national retrospective data linkage study. *BMC Medicine*, 17(1), 2. doi: 10.1186/s12916-018-1234-0 30630493 PMC6329132

[3] Beecham L. (1999). Missed GP appointments cost NHS money. *BMJ*, 319(7209), 536.10463888

[4] Gucciardi E. (2008). A systematic review of attrition from diabetes education services: strategies to improve attrition and retention research. *Canadian Journal of Diabetes*, 32 *(*1*)*, 53–65.

[5] Murdock A., Rodgers C., Lindsay H. and Tham T. C. K. (2002). Why do patients not keep their appointments? Prospective study in a gastroenterology outpatient clinic. *Journal of the Royal Society of Medicine*, 95 *(*6*)*, 284–286. doi: 10.1258/jrsm.95.6.284 12042374 PMC1279909

[6] Hallsworth M., Berry D., Sanders M., Sallis A., King D., Vlaev I. and Darzi A. (2015). Stating appointment costs in SMS reminders reduces missed hospital appointments: findings from two randomised controlled trials. *PLOS ONE*, 10 *(*9*)*, e0137306. doi: 10.1371/journal.pone.0137306 26366885 PMC4569397

[7] Hasvold P. E. and Wootton R. (2011). Use of telephone and SMS reminders to improve attendance at hospital appointments: a systematic review. *Journal of Telemedicine and Telecare*, 17(7), 358–364. doi: 10.1258/jtt.2011.110707 21933898 PMC3188816

[8] Ellis D. A., McQueenie R., McConnachie A., Wilson P and Williamson A. E. (2017). Demographic and practice factors predicting repeated non-attendance in primary care: A national retrospective cohort analysis. *The Lancet Public Health*. 2 *(*12*)*, e551–e559 doi: 10.1016/S2468-2667(17)30217-7 29253440 PMC5725414

[9] Ellis D. A. and Jenkins R. (2012). Weekday affects attendance rate for medical appointments: Large-scale data analysis and implications. *PLOS ONE*, 7 *(*12*)*: e51365 doi: 10.1371/journal.pone.0051365 23272102 PMC3521765

[10] Menendez M. E. and Ring D. (2015). Factors associated with non-attendance at a hand surgery appointment. *Hand*, 10(2), 221–226. doi: 10.1007/s11552-014-9685-z 26034434 PMC4447667

[11] Nayor J., Maniar S. and Chan W. W. (2017). Appointment-keeping behaviors and procedure day are associated with colonoscopy attendance in a patient navigator population. *Preventive medicine*, 97, 8–12. doi: 10.1016/j.ypmed.2016.12.022 28024864

[12] Ellis D. A., McQueenie R., Williamson A. E., & Wilson P. (2020). Missed appointments in health care systems: A national retrospective data linkage project. *SAGE Research Methods Cases*.

[13] Kuhn K. M. (2016). The rise of the “gig economy” and implications for understanding work and workers. *Industrial and Organizational Psychology*, 9 *(*1*)*, 157–162.

[14] Ellis D. A. Wiseman R. and Jenkins R. (2015). Mental representations of weekdays. *PLOS ONE*. 10 *(*8*)*: e0134555 doi: 10.1371/journal.pone.0134555 26288194 PMC4544878

[15] Paterson B. L. Charlton P. and Richard S. (2010). Non-attendance in chronic disease clinics: a matter of non-compliance? *Journal of Nursing and Healthcare of Chronic Illness*, 2(1), 63–74.

[16] Lawson V. L., Lyne P. A., Harvey J. N. and Bundy C. E. (2005). Understanding why people with type 1 diabetes do not attend for specialist advice: a qualitative analysis of the views of people with insulin-dependent diabetes who do not attend diabetes clinic. *Journal of Health Psychology*, 10(3), 409–423. doi: 10.1177/1359105305051426 15857871

[17] Lacy N. L., Paulman A., Reuter M. D. and Lovejoy B. (2004). Why we don’t come: patient perceptions on no-shows. *The Annals of Family Medicine*, 2 *(*6*)*, 541–545. doi: 10.1370/afm.123 15576538 PMC1466756

[18] Sanders J. G. and Jenkins R. (2016). Weekly Fluctuations in Risk Tolerance and Voting Behaviour. *PLOS ONE*, 11(7), e0159017. doi: 10.1371/journal.pone.0159017 27392020 PMC4938543

